# Using Mixed Reality Headsets to Deliver Remote Bedside Teaching During the COVID-19 Pandemic: Feasibility Trial of HoloLens 2

**DOI:** 10.2196/35674

**Published:** 2022-05-17

**Authors:** Arun Sivananthan, Aurelien Gueroult, Geiske Zijlstra, Guy Martin, Aravindhan Baheerathan, Philip Pratt, Ara Darzi, Nisha Patel, James Kinross

**Affiliations:** 1 Institute of Global Health Innovation Imperial College London London United Kingdom; 2 Department of Gastroenterology and Hepatology Imperial College Healthcare NHS Trust London United Kingdom; 3 Department of Academic Public Health Imperial College London London United Kingdom; 4 Department of Surgery and Cancer Imperial College London London United Kingdom; 5 Department of Neurology Imperial College Healthcare NHS Trust London United Kingdom; 6 Medical iSight Corporation London United Kingdom

**Keywords:** mixed reality, remote learning, HoloLens, bedside teaching, COVID-19, personal protective equipment, digital education, medical education, e-learning, protection, feasibility, medical student, virtual reality

## Abstract

**Background:**

COVID-19 has had a catastrophic impact in terms of human lives lost. Medical education has also been impacted as appropriately stringent infection control policies precluded medical trainees from attending clinical teaching. Lecture-based education has been easily transferred to a digital platform, but bedside teaching has not.

**Objective:**

This study aims to assess the feasibility of using a mixed reality (MR) headset to deliver remote bedside teaching.

**Methods:**

Two MR sessions were led by senior doctors wearing the HoloLens headset. The trainers selected patients requiring their specialist input. The headset allowed bidirectional audiovisual communication between the trainer and trainee doctors. Trainee doctor conceptions of bedside teaching, impact of the COVID-19 pandemic on bedside teaching, and the MR sessions were evaluated using pre- and postround questionnaires, using Likert scales. Data related to clinician exposure to at-risk patients and use of personal protective equipment (PPE) were collected.

**Results:**

Prequestionnaire respondents (n=24) strongly agreed that bedside teaching is key to educating clinicians (median 7, IQR 6-7). Postsession questionnaires showed that, overall, users subjectively agreed the MR session was helpful to their learning (median 6, IQR 5.25-7) and that it was worthwhile (median 6, IQR 5.25-7). Mixed reality versus in-person teaching led to a 79.5% reduction in cumulative clinician exposure time and 83.3% reduction in PPE use.

**Conclusions:**

This study is proof of principle that HoloLens can be used effectively to deliver clinical bedside teaching. This novel format confers significant advantages in terms of minimizing exposure of trainees to COVID-19, reducing PPE use, enabling larger attendance, and delivering convenient and accessible real-time clinical training.

## Introduction

The initial outbreak of SARS-CoV-2 in Wuhan, China, in December 2019 developed into a global pandemic with an unavoidable collateral impact on postgraduate medical education. As of March 6, 2022, almost 446 million confirmed cases of COVID-19 and over 6 million deaths have been reported in 226 countries [1]. Further to this catastrophic impact measured in human lives, the pandemic has engendered major organizational change in health care systems and had significant economic impacts [2].

Bedside teaching plays an important role in medical education for postgraduate doctors with evidence of benefit to trainees, patients, and clinicians [3]. Despite increasingly sophisticated and capable diagnostic investigations, good history taking and clinical examination remain a core part of the diagnostic process [4].

Stringent infection control policies were introduced in hospital settings with the aim of preventing nosocomial spread of infection and protecting health care workers. These involved use of personal protective equipment (PPE), minimizing clinician contact, and social distancing. These necessary precautions had an impact on traditional forms of medical education. Social distancing has precluded students and junior trainees from gathering for teaching and clinical rotations have been cancelled.

The clinical environment in which students would usually acquire practical skills and form their professional identity was severely restricted by infection control policies during COVID-19 [5]. In a survey of 152 junior doctors in May 2020 at a UK teaching hospital, only 21.1% of participants felt that their educational needs were being met during the COVID-19 period. Learning opportunities have become fewer as operations have been cancelled and clinics transitioned to telemedical modalities. Despite this, bedside teaching has not undergone the same degree of digital transformation. However, novel telemedicine technologies may provide a solution to this challenge.

Augmented reality (AR) merges the virtual world and real world—the “display of an otherwise real environment is augmented by means of virtual (computer graphic) objects” [6]. Mixed reality (MR) is a subtype of AR where these digital objects can be manipulated and interacted with in real time as if part of the real world.

HoloLens 2 is an MR head-mounted device developed and marketed by Microsoft Corp. The headset combines several types of sensors to provide a true “heads-up display” with the ability to place virtual “hologram” objects within the user’s visual field. It also permits live bidirectional communication via video, MR composites, and voice with multiple remote users. In a clinical setting, this allows the wearer of the device to share their clinical interaction remotely with multiple connected users as well as display and manipulate “holographic” images (within the real environment) that can also be collaboratively interacted with by the remote users.

In simple terms, this allows the patient interaction to be observed remotely by multiple trainees; in addition, participants can display and collaboratively interact with a multitude of clinically relevant “holograms” such as blood results, radiological images, or educational figures [7]. This remote yet intrasituational learning confers specific benefits in the context of the COVID-19 pandemic in terms of reducing risk of contagion and economizing PPE, especially for “nonessential” tasks.

HoloLens and similar MR devices have already been trialed in medical education, specifically for teaching anatomy [8,9]. A recent pilot study at this center demonstrated that the use of HoloLens during the COVID-19 pandemic for direct clinical care reduced time exposure of staff caring for patients with COVID-19 by 51.5%, with an 83.1% reduction in PPE use [10].

The aim of this study was to assess the feasibility of using a HoloLens device and the MR environment to deliver remote bedside teaching to trainee doctors to support the delivery of higher-quality care during the COVID-19 pandemic.

## Methods

### Overview

The MR sessions were led by senior specialty registrars (the clinical trainers). The clinical trainers were senior specialty trainees who regularly delivered bedside teaching prior to the introduction of social distancing rules.

Patients with demonstrable pathology or interesting histories, who would normally have been recruited for in-person bedside teaching, were selected. Patients were required to have the capacity to consent to use of the HoloLens headset and be able to communicate with the clinician. Social distancing would normally preclude junior doctors from observing these educational consultations. HoloLens was therefore used opportunistically to allow trainees to virtually attend while adhering to infection control rules.

Trainers received a briefing and had 10 minutes of practice time using the HoloLens device. The practice time included completion of the inbuilt training program by the trainers. Following this, a simulated call was set up to allow the trainer to familiarize themselves with the device.

**Figure 1:**
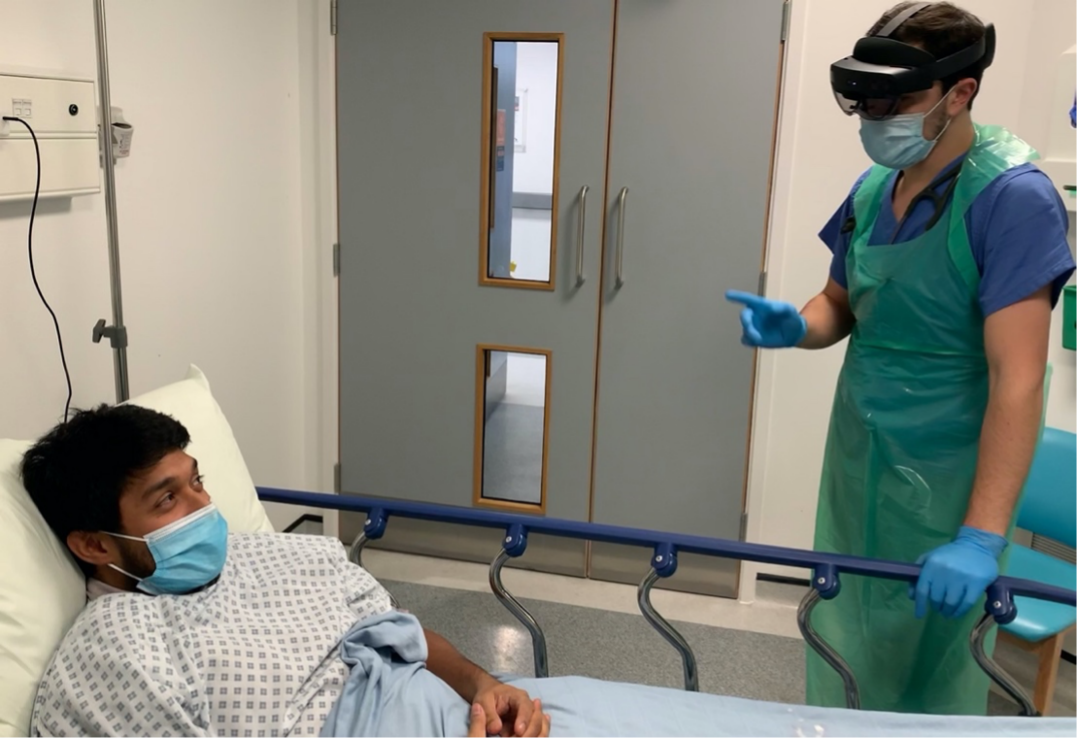
HoloLens used as part of personal protective equipment (simulated using staff for patient confidentiality, presented with consent by Jabed Ahmed and authors AS and AG).

The HoloLens headset, in collaboration with the local infection prevention and control team, was incorporated as personal protective eye protection (Figure 1) with a decontamination protocol [10].

### Ethical Considerations

Local institutional registration and approval was obtained, and data governance, infection prevention, and control procedures were agreed upon prior to the commencement of the project. No additional ethical approval was required as the project was conducted as a technology-led quality improvement project under the supervision of the institutional quality improvement team. No patient identifying images are provided (situations were simulated by authors and consent for the publication of the images was given). Informed consent was received from the patients by the clinical trainer to allow trainee remote observation prior to the remote session.

### Remote Bedside Teaching Sessions

Approximately 4-6 trainee doctors participated via a secure, two-factor authentication video link system (Microsoft Teams) and connected to the session on a trusted computer with webcam enabled.

**Figure 2:**
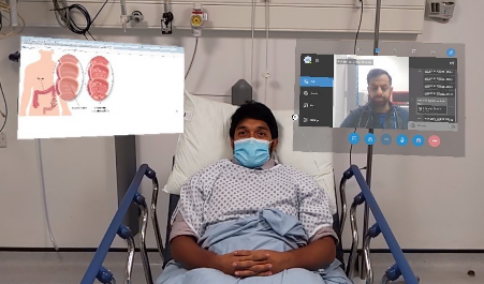
First-person view from the trainer wearing the HoloLens and the same view seen by remote attendee. Video and audio bidirectional communication with trainees occurs through the right screen. Relevant clinical images are shared on the left screen. Both "holograms" can be manipulated by hand gestures (simulated using staff for patient confidentiality, presented with consent by Jabed Ahmed and authors AS and AG).

On entering the patient’s room, the trainer placed a fixed “hologram” of the trainee doctors “attending” adjacent to the patient, allowing bidirectional video and audio communication between the trainer and trainee doctors during the consultation (Figure 2). This was also supported by a chat function beside the “hologram.” This allowed the trainees to communicate in two ways: by voice or by typing via the chat function, which appeared on the screen of the trainer, where the trainee doctors could type questions and responses. The trainee doctors were also able to select radiological images and educational figures to superimpose on the real view at the trainer’s request, which would be visible for all attendees.

Two sessions were carried out. The first session reviewed a 28-year-old male presenting with bloody diarrhea and a likely flare of ulcerative colitis; this session was led by a gastroenterology specialist trainee. The second session reviewed a 62-year-old presenting with double vision and a likely third nerve palsy; this session was led by a neurology specialist trainee. Both trainers were due to clinically review the patient. Thus, these reviewers were used opportunistically for teaching so as not to increase exposure risk.

### Ward Round Format

Gagné’s [11] principles of instruction, Ker et al’s [12] six questions for planning teaching ward rounds, and Abdool and Bradley’s [13] work on improving medical teaching rounds were used to design the format for the HoloLens teaching ward round. Key parts of both papers were used to inform the structure of the session.

Prior to entering the patient’s room, aims for the session were established by the group. The trainer then presented the initial vignette of the case, moving on to an open discussion of potential history questions and relevant clinical examination. This allowed the initial principles Gagne described to be fulfilled (ie, “gain attention of students, stimulate recall of prior learning, and provide learning guidance” [11]).

The instructor then entered the room. A history and clinical examination was taken, with participants encouraged to ask questions verbally or using the Teams chat function to most closely simulate an in-person bedside teaching session. Discussion of the case was aided by superimposition of relevant images as additional “holograms,” such as endoscopy images demonstrating the patient’s inflamed mucosa and diagrams showing the anatomy of the colon.

The session was then concluded away from the bedside. Questions were “scaled up the hierarchy,” as described by Abdool and Bradley [12], where simpler questions were asked to more junior members of the team and harder questions to more senior members to allow for the broad spread of attendees. Key learning points were discussed and documented collaboratively using the chat function during the reflection phase.

### Outcomes

Trainee doctor conceptions of bedside teaching, impact of the COVID-19 pandemic on bedside teaching, and technical success of the session were evaluated anonymously using pre- and postround questionnaires using Likert scales (from 1-7) and open questions.

Cumulative data regarding exposure to at-risk patients and use of PPE were calculated for the virtual sessions against the projected exposure and PPE use for in-person teaching.

## Results

A total of 24 participants answered the presession questionnaire, of which 19 were junior doctors and 4 were specialist trainees. Table 1 summarizes their responses. Participants strongly agreed that bedside teaching is key to educating clinicians (median 7, IQR 6-7). It was also apparent that bedside teaching had been severely affected by the COVID-19 pandemic, becoming a rarity (median 2, IQR 2-4).

Session 1 had 6 participants and session 2 had 4 participants, all of whom had answered the prequestionnaire. There were 3 interim foundation doctors, 5 foundation year 1 doctors, and 1 registrar. Table 2 summarizes their responses to the postteaching questionnaire. The first session was affected by a loose microphone connection and this was reflected by participants strongly disagreeing (median 2.5, IQR 1.25-3) that they were able to see and hear the patient-clinician interaction as if they had been present in person. In the second session, with the issue rectified, the quality of video and audio allowed respondents to appreciate subtle clinical signs and most strongly agreed that they felt like they were physically present (median 7, IQR 6.75-7). Feedback regarding session engagement, usefulness, and quality was positive across both sessions.

Both teaching sessions were undertaken with at-risk patients with COVID-19 in the rooms. Session 1 and 2 lasted 26 and 33 minutes, respectively. Cumulative clinician exposure to at-risk patients was 59 minutes versus 288 minutes had bedside teaching occurred in person. This was equivalent to a 79.5% reduction in exposure. Furthermore, 10 pieces of disposable PPE (gown, apron, gloves, and mask) alongside the HoloLens were used to facilitate teaching versus 60 pieces of disposable PPE had bedside teaching occurred in person. This was equivalent to an 83.3% reduction in PPE use.

**Table 1:** Respondents’ scores to the presession questionnaire.

Prequestionnaire questions	Respondents’ scores (n=24), median (IQR)
In my past experience, teaching occurs on ward rounds	4 (3-4)^a^
In my recent experience, during the COVID-19 period, teaching occurs on ward rounds	3 (2-3.25)^a^
Bedside teaching is key to educating clinicians	7 (6-7)^b^
I have had bedside teaching during the COVID-19 period	2 (2-4)^a^
I feel able to ask the senior clinician educational questions during ward rounds	4.5 (3-5.25)^b^

^a^Denotes Likert scale scores ranging from 1=never to 7=every day.

^b^Denotes Likert scale scores ranging from 1=strongly disagree to 7=strongly agree.

**Table 2:** Respondents’ scores to the postsession questionnaire.

Postquestionnaire questions	Respondents’ scores, median (IQR)^a^		
	First session (n=6)	Second session (n=4)	Pooled (n=10)
I was able to see and hear the patient-clinician interaction like I was in the room	2.5 (1.25-3)	7 (6.75-7)	4.5 (2.25-6.75)
The teaching was relevant to me	6.5 (6-7)	6.5 (5.75-7)	6.5 (6-7)
I found the clinician leading the ward round engaging as a teacher	7 (6.25-7)	7 (7-7)	7 (7-7)
I felt able to ask the clinician leading the ward round educational questions	7 (6.25-7)	6.5 (5.75-7)	7 (6-7)
I felt like my questions were answered	7 (6.25-7)	6.5 (6-7)	7 (6-7)
The session was helpful to my learning	5.5 (3.5-6.75)	6.5 (6-7)	6 (5.25-7)
In my opinion, the use of HoloLens in this context is worthwhile	5.5 (3.5-6.75)	6.5 (6-7)	6 (5.25-7)

^a^All answers presented as Likert scale scores ranging from 1=strongly disagree to 7=strongly agree.

## Discussion

### Principal Findings

This study is proof of principle that MR headsets can be used to deliver clinical bedside teaching with at-risk patients while reducing exposure risk and PPE use.

Objectively, there was a reduction in exposure and PPE use by clinicians. Subjectively, trainees agreed that they were able to ask questions, had their questions answered, and found the session helpful to their learning. Overall, all trainees agreed that the use of HoloLens was worthwhile in this context.

In the first session, the microphone was not plugged in correctly, which likely explains why trainees disagreed with the statement that they could hear the patient-clinician interaction as if in the room. This improved in the second session, when the microphone was appropriately plugged in and all trainees agreed with the statement.

A steep learning curve was observed, with trainers able to use the headset confidently after 1-2 practice sessions. This novel format may confer significant advantages in terms of minimizing exposure of trainees and medical students to SARS-CoV-2, saving PPE, enabling much larger attendance than possible at traditional bedside teaching, creating an environment that is less intimidating for the patient, and enabling real-time application of learning to a clinical context (eg, by augmenting the headset view with educational figures).

A potential limitation of remote bedside teaching is the inability of the trainee to clinically examine the patient or appreciate signs themselves. The pandemic has necessitated such an approach as remote teaching was the best available surrogate to in-person teaching. It is unclear, however, if there is a difference in the educational impact of MR remote versus in-person bedside teaching. Similarly, the clinical cases chosen had signs that were appreciable remotely but some clinical signs require tactile feedback (eg, examining limb tone during a neurological examination, or auscultation for cardiac murmurs). On this latter point, we note that there exist digital stethoscopes that would allow sharing of auscultation findings and these are potentially compatible with a HoloLens.

This study itself was purely a feasibility trial and therefore it is difficult to comment on much beyond the technical feasibility to deliver a remote MR bedside teaching session.

Further research would be useful to ascertain the educational effectiveness of such an intervention using measures beyond subjective feedback. It is important to note that telemedicine is available in simpler and more accessible formats and the additional potential of the MR component, specifically to share educational figures and radiological images at the bedside, needs to be further investigated.

The pandemic has fostered innovation, with remote teaching becoming a normal part of medical postgraduate training. Remote training has demonstrated several potential benefits independent of the pandemic and social distancing. Reduction in travel is time-saving for trainees and has a positive impact on the carbon footprint. The digital nature of remote teaching allows for recording and increases accessibility, so trainees can watch at their own convenience and not miss out due to clinical commitments. Remote bedside teaching itself allows opportunistic examination of rare clinical signs normally limited to physical attendees. Remote bedside teaching allows greater access to these rare and valuable learning opportunities.

The ability to create a virtual classroom at the bedside where learners can remotely attend a bedside clinical teaching session and relevant results images can be shared, interacted with, and discussed is a novel evolution of the traditional bedside teaching format. Its benefits in a pandemic situation are clear but its applicability outside of this context has potential and will need further investigation.

### Conclusions

MR bedside teaching is technologically feasible and acceptable using the HoloLens platform. This confers significant benefit, allowing bedside teaching to continue while complying with stringent and necessary infection prevention strategies during the COVID-19 pandemic. Beyond the pandemic, there may indeed be additional benefits including the convenience of remote attendance and the introduction of educational “holograms” to the bedside. Further research into this exciting educational platform is warranted.

## Abbreviations

AR: augmented reality
MR: mixed reality
PPE: personal protective equipment
